# A Fabry–Perot Sensor with Cascaded Polymer Films Based on Vernier Effect for Simultaneous Measurement of Relative Humidity and Temperature

**DOI:** 10.3390/s23052800

**Published:** 2023-03-03

**Authors:** Teng Zhang, Qun Han, Zhizhuang Liang, Junfeng Jiang, Zhenzhou Cheng

**Affiliations:** 1 School of Precision Instrument and Opto-Electronics Engineering, Tianjin University, Tianjin 300072, China; 2 Key Laboratory of Opto-Electronics Information Technology of the Ministry of Education, Tianjin University, Tianjin 300072, China

**Keywords:** Fabry–Perot interferometer, cascaded polymer films, optical fiber sensor, optical Vernier effect

## Abstract

In this paper, fiber sensor based on Vernier effect for simultaneous measurement of relative humidity (RH) and temperature is proposed. The sensor is fabricated by coating two kinds of ultraviolet (UV) glue with different refractive indexes (RI) and thicknesses on the end face of a fiber patch cord. The thicknesses of two films are controlled to generate the Vernier effect. The inner film is formed by a cured lower-RI UV glue. The exterior film is formed by a cured higher-RI UV glue, of which thickness is much thinner than the inner film. Through the analysis of the Fast Fourier Transform (FFT) of the reflective spectrum, the Vernier effect is formed by the inner lower-RI polymer cavity and the cavity composed of both polymer films. By calibrating the RH and temperature response of two peaks on the envelope of the reflection spectrum, simultaneous measurements of RH and temperature are realized by solving a set of quadratic equations. Experimental results show that the highest RH and temperature sensitivities of the sensor are 387.3 pm/%RH (in 20%RH to 90%RH) and −533.0 pm/°C (in 15 °C to 40 °C), respectively. The sensor has merits of low cost, simple fabrication, and high sensitivity, which makes it very attractive for applications that need to simultaneously monitor these two parameters.

## 1. Introduction

Relative humidity (RH) and temperature are the two most important environmental parameters in many applications, such as monitoring the living conditions of manned spacecrafts, controlling specific chemical reactions, and managing certain hazardous article warehouses. Compared with electronic hygrometers, optical fiber humidity sensors have the advantages of electromagnetic interference resistance, high sensitivity, quick response and so on [1,2,3]. Several kinds of fiber RH sensors have been reported, such as those based on tapered fiber [4,5], Mach–Zehnder interferometers(MZI) [6,7,8], Fabry–Perot interferometers (FPI) [9,10], optical fiber Bragg gratings [11,12], and microfiber knot resonators [13]. However, these RH sensors lack the ability to measure the temperature simultaneously. Relative humidity is defined as the percentage of current water vapor pressure to the saturated water vapor pressure at the same temperature [14]. This means that in order to ensure the accuracy of the RH measurement, the corresponding temperature must be measured at the same time. Moreover, temperature can also affect the accuracy of the traditional RH optical fiber sensors through the thermo-optic effect of the fiber material. In 2022, Li et al. [15] proposed an optical fiber sensor based on cascaded C-shaped Fabry–Perot interferometers to measure RH and temperature simultaneously. They added two kinds of solid polymer materials to the C-shaped fiber interferometers for dual parameters sensing. The reported sensor had RH sensitivities of −0.128 nm/%RH and 0.038 nm/%RH in the range of 20%RH to 45%RH, and temperature sensitivities of 0.022 nm/°C and −0.722 nm/°C in the temperature range of 15 °C to 45 °C. However, the measurement range of RH was small (from 20%RH to 45%RH), and the RH sensitivity of the sensor was low. In 2021, Min et al. [16] coated polymer optical fiber Bragg gratings (POFBGs) with an UV cure adhesive to obtain chirped Bragg gratings for multiparameter sensing. In the reported experiment, the RH sensitivities of two distinct sensing active areas were 11.8 ± 0.2 pm/%RH and 13.7 ± 0.5 pm/%RH, while the strain sensitivities were 0.719 ± 0.020 pm/µε and 0.122 ± 0.005 pm/µε. However, in the thermal characterization, the relationship between the wavelength of the peak and temperature is not monotonic. As a result, the sensor lacked the ability to measure RH and temperature simultaneously. Meanwhile, the RH sensitivity of the sensor was low compared with other dual parameters sensors. In 2020, Tong et al. [17] presented a compact system for simultaneous measurement of RH and temperature, which was composed of a FPI and an MZI. The MZI structure was formed by core-offset splicing a 200 µm SMF between two 10 cm SMFs with a core offset of 62.8 µm. The core offset region was coated by RH-sensitive material GQDs-PVA. The RH sensitivity of the sensor was −0.132 nm/%RH, and the temperature sensitivity was 0.37 nm/°C. However, the sensor was fragile since the large core offset structure. In 2018, Wang et al. [18] presented a simultaneous measurement sensor for RH and temperature, which was composed of a fiber Bragg grating (FBG) and an FPI. The FPI was composed of a single-mode fiber, a hollow capillary and a polyimide film filled at the tip of the hollow capillary. By splicing a FBG, which is sensitive to temperature, the sensor can measure RH and temperature simultaneously. However, the fabrication was complex. Additionally, the temperature and RH sensitivities of the measurement system were 9.98 pm/°C and 22.07 pm/%RH, respectively. The low sensitivities made the sensor difficult to be widely applied.

Due to the ability of sensitivity to environmental parameters, many polymeric materials are used to measure RH, temperature or other parameters. Polymer FPI is a common structure in optical fiber sensors. In 2014, Su et al. [10] proposed a RH sensor with a structure of FPI. The FPI was formed by coating a PVA film on the end face of a SMF. In the reported experiment, the RH sensitivity of the FPI sensor was 70 pm/%RH, in the range of 7%RH to 91.2%RH. In 2019, Vaz et al. [9] proposed a FPI sensor for RH measurement. The FP cavity was formed by a hollow silicon coated with a PVDF thin film. The RH sensitivity of the sensor was 32.54 pm/%RH. In 2022, Wang et al. [19] proposed a FP cavity with acrylate AB glue coated on the end face of a glass tube for RH sensing. The RH sensitivity was 80.68 pm/% RH. These traditional polymeric FPI sensors for RH sensing had the disadvantage of low sensitivity.

In recent years, the Vernier effect has been utilized to enhance the sensitivity of optical fiber sensors. This strategy utilizes the superimposition of two or more similar interferometers to improve the sensitivity of the sensor. For example, in 2019, Hou et al. [20] proposed a fiber temperature sensor based on cascaded Fabry–Perot interferometers with one cavity filed with PDMS. By utilizing the Vernier effect, a high temperature sensitivity of 17.758 nm/°C was achieved. In 2021, a similar structure was proposed for magnetic field sensing [21] with a FPI cavity filled with magnetic fluid. Due to the enhancement of the Vernier effect, a high sensitivity of 1.02602 nm/Gs was realized in the range of 118.768 Gs to 166.261 Gs.

In this paper, a RH and temperature simultaneous measurement sensor with a very simple structure is proposed. The sensor was fabricated by curing two kinds of ultraviolet (UV) glue with different refractive indexes (RIs) to form two layers of polymer films with different thicknesses on the end face of a fiber patch cord. Thus, a cascaded Fabry–Perot interferometer (FPI) was formed. The change of the surrounding RH only influences the RI of the exterior layer, whereas temperature change affects the RIs of both layers. By solving a set of quadratic equations, RH and temperature can be demodulated simultaneously. The Vernier effect has also been utilized to enhance the sensitivity [20,21,22].

## 2. Sensor Fabrication and Working Principle

**Figure 1:**
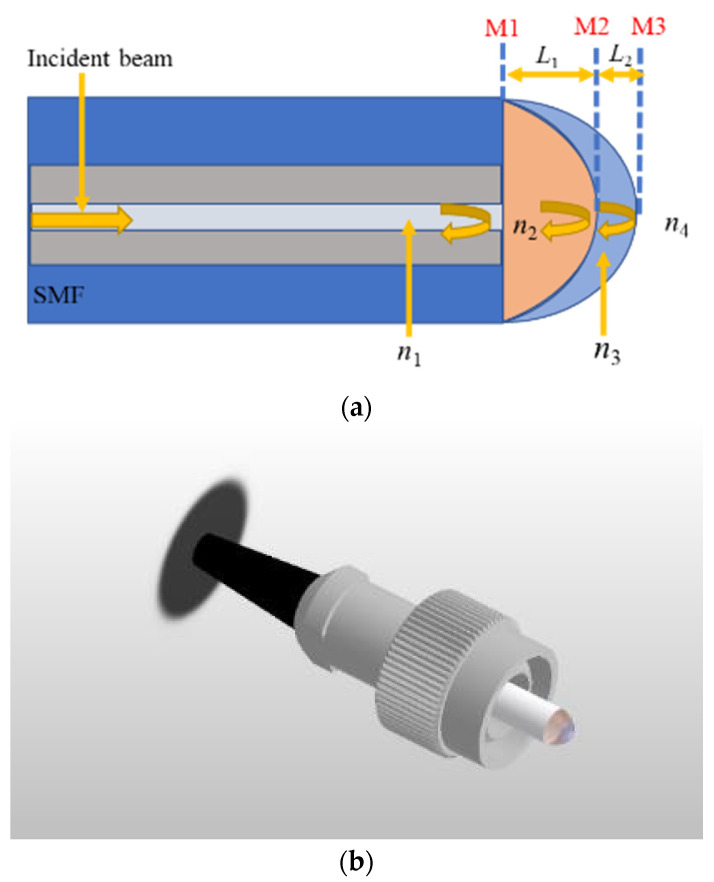
(**a**) Schematic diagram and (**b**) 3D illustration of proposed sensor.

**Figure 2:**
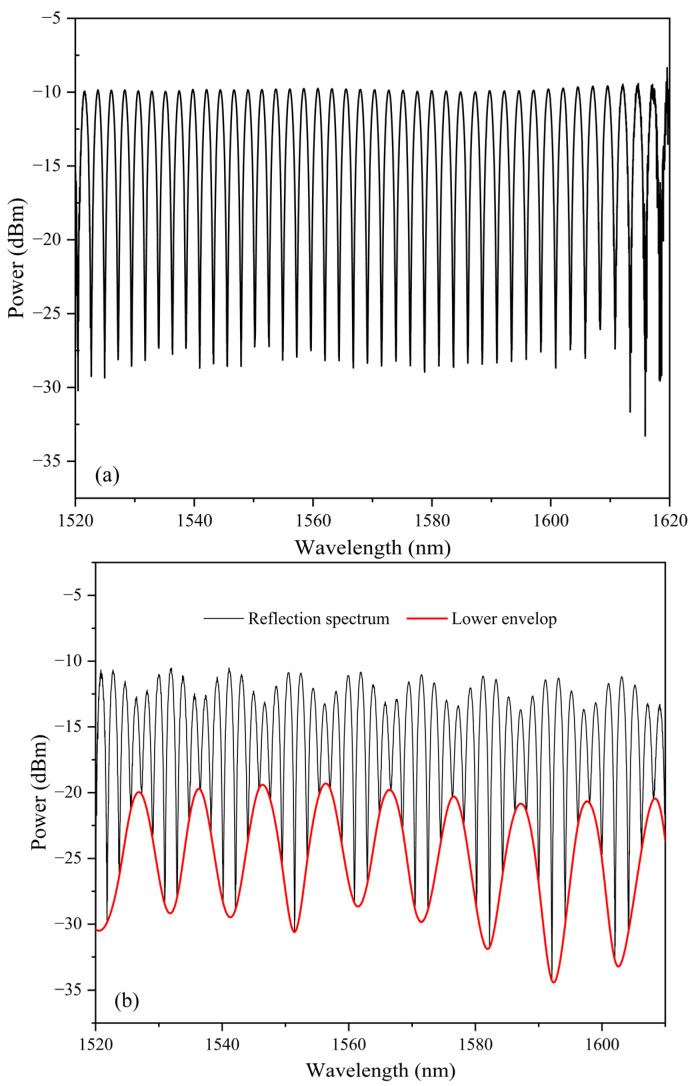
The reflection spectrum after (**a**) the first layer and (**b**) the second layer was cured.

The schematic diagram and a 3D illustration of the sensor are shown in Figure 1. Two polymer films were fabricated directly on the end face of a polished fiber patch cord with an FC connector. The fiber in the patch cord is a standard singlemode fiber (SMF). In order to ensure an RI difference as large as possible and optimize the visibility of the interference spectrum, the UV glues of the first layer and second layer were choose to be PC 373 LD (Luvantix ADM Inc., Daejeon, North Korea) and the NOA 144 (Norland Products Inc., Jamesburg, NJ, USA). The corresponding RIs are $n_{2}=1.373$ and $n_{3}=1.440$, respectively. The fabrication process is as follows: 

Firstly, a drop of the low RI glue was dropped on a slide. Then, the end face of the fiber patch cord was shallowly dipped into it vertically. A hemispherical film would be formed on the end face after the fiber was moved up, due to surface tension of the glue. During the whole process, the reflection spectrum was monitored in real-time by an optical spectrum analyzer (ADVANTEST Q8384). If the thickness of the film needs to be slightly adjusted, the connector can be turned bottom-up. Under the combined action of the viscosity of the glue and gravity, the thickness of the film will change slowly. When the desired spectrum was observed on the OSA, the UV light source was tuned on to cure the glue quickly. The reflection spectrum after the curing of the first layer is shown in Figure 2a. It can be seen that the peak-valley difference is about 20 dB. 

The second layer was coated with the same procedure as NOA 144. After coating the second layer, the reflection spectrum is shown in Figure 2b. The envelop is formed by the Vernier effect of the cascaded FPIs.

As depicted in Figure 1a, Fresnel reflections from the three interfaces (M1, M2, and M3) form three FPIs, i.e., FPI1 formed by M1 and M2, FPI2 formed by M2 and M3, and FPI3 formed by M1 and M3. The corresponding cavity lengths of the FPIs are $L_{1}$, $L_{2}$, and $L_{3}=L_{1}+L_{2}$, respectively. The electric field of the reflected beam can be expressed as follows [21]:

(1) $$E=E_{0}[\sqrt{R_{1}}+Ae^{-j2\varphi_{1}}+Be^{-j2(\varphi_{1}+\varphi_{2})}]$$

where $E_{0}$ is the electric field of the input light beam, $\varphi_{1}$ and $\varphi_{2}$ are the round-trip phase shift of FPI1 and FPI2, respectively, which can be expressed as:

(2) $$\begin{array}{l} \varphi_{1}=\frac{2\pi n_{2}L_{1}}{\lambda },\\ \varphi_{2}=\frac{2\pi n_{3}L_{2}}{\lambda }, \end{array}$$

and [23]

(3) $$\begin{array}{l} A=(1-\varepsilon_{1})(1-R_{1})\sqrt{R_{2}},\\ B=(1-\varepsilon_{1})(1-\varepsilon_{2})(1-R_{1})(1-R_{2})\sqrt{R_{3}}, \end{array}$$

where $\varepsilon_{1}$ and $\varepsilon_{2}$ are the transmission loss of FPI1 and FPI2, respectively, and $R_{1}$, $R_{2}$, and $R_{3}$ are the reflection coefficients of M1, M2, and M3, respectively. They can be expressed as:

(4) $$\begin{array}{l} \;R_{1}={\left(\frac{n_{1}-n_{2}}{n_{1}+n_{2}}\right)}^{2},\\ R_{2}={\left(\frac{n_{2}-n_{3}}{n_{2}+n_{3}}\right)}^{2},\\ R_{3}={\left(\frac{n_{3}-n_{4}}{n_{3}+n_{4}}\right)}^{2}, \end{array}$$

where $n_{1}$, $n_{2}$, $n_{3}$ and $n_{4}$ are the refractive indices of the core of the SMF, the polymer film with a lower refractive index, the polymer film with a higher refractive index, and air, respectively, as shown in Figure 1. 

The relative intensity of the reflected beam can be expressed as:

(5) $$\begin{array}{r} I={\left|\frac{E}{E_{0}}\right|}^{2}=R_{1}+A^{2}+B^{2}+2\sqrt{R_{1}}B\cos\left[2\left(\varphi_{1}+\varphi_{2}\right)\right]+\\ 2\sqrt{R_{1}}A\cos(2\varphi_{1})+2AB\cos(2\varphi_{2}) \end{array}$$

The free spectrum range (*FSR*) of a single FPI can be expressed as:

(6) $$FSR=\frac{\lambda^{2}}{2nL}=\frac{1}{f}$$

where f is the frequency of the interference spectrum, which is the reciprocal of the free spectrum range. 

**Figure 3:**
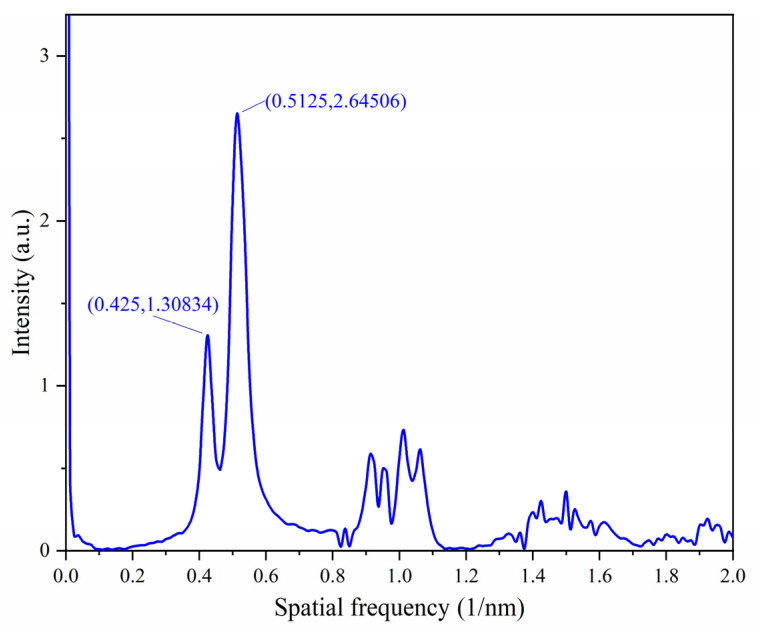
FFT of the reflection spectrum.

The FFT of the reflection spectrum is shown in Figure 3. It can be seen that the reflected beam is the superposition of two components with a small difference in frequency. The corresponding cavity lengths are 369.9 µm and 442.5 µm. This means that the reflection spectrum is mainly composed of the FPI1 and FPI3 [21]. The length of the FPI2, i.e., *L*_2_ = 72.6 µm. Therefore, the *FSR* of the periodic envelope can be expressed as:

(7) $$FSR_{envelope}=\frac{FSR_{1}\cdot FSR_{3}}{\left|FSR_{1}-FSR_{3}\right|}$$

where

(8) $$FSR_{1}=\frac{\lambda^{2}}{2n_{2}L_{1}}$$

is the *FSR* of FPI1;

(9) $$FSR_{3}=\frac{\lambda^{2}}{2n_{2}L_{1}+2n_{3}L_{2}}$$

is the *FSR* of FPI3. Compared with a single FPI3, the sensitivity is amplified *M* times due to the Vernier effect, where *M* can be expressed as follows:

(10) $$\begin{array}{c} M=\frac{FSR_{1}}{\left|FSR_{1}-FSR_{3}\right|}\\ =1+\frac{n_{2}L_{1}}{n_{3}L_{2}}. \end{array}$$

Clearly, *M* is determined by the ratio of the optical path length of FPI1 and FPI2. For the sensor fabricated previously, *M* is about 5.86. For FPI3, the center wavelength of the *m*th dip of the interference fringes can be expressed as:

(11) $$\lambda_{m}=\frac{4\left(n_{2}L_{1}+n_{3}L_{2}\right)}{2m+1}$$

Because FPI1 is insensitive to RH, the RH shifts the spectrum mainly through the change of $n_{3}$ and $L_{3}$. The humidity sensitivity $S_{H}$ can be expressed as follows:

(12) $$S_{H}=M\lambda_{m}\left(\frac{\Delta n_{3}\cdot L_{2}+n_{3}\cdot \Delta L_{2}}{n_{2}L_{1}+n_{3}L_{2}}\right).$$

The RH sensitivity is a combined effect of the RI and length change of the FPI2, after the film absorbs the water molecules. The temperature $S_{T}$ can be deduced in a similar way, but now both cavities are sensitive to temperature. It can be expressed as follows:

(13) $$S_{T}=M\lambda_{m}\left(\frac{\Delta n_{2}\cdot L_{1}+n_{2}\cdot \Delta L_{1}+\Delta n_{3}\cdot L_{2}+n_{3}\cdot \Delta L_{2}}{n_{2}L_{1}+n_{3}L_{2}}\right).$$

The temperature sensitivity is resulted from the thermal expansion and thermal optical effect of the polymer films.

## 3. Experimental Results and Discussion

**Figure 4:**
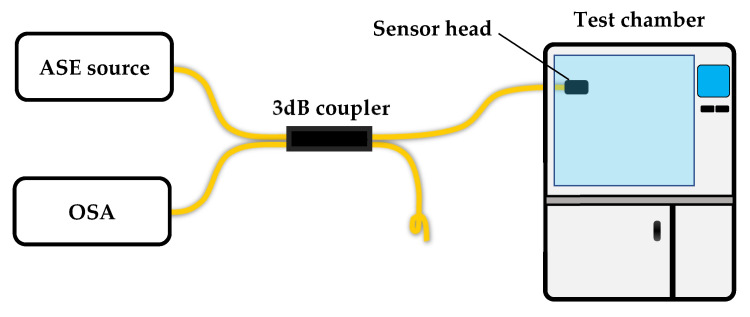
Schematic diagram of the experimental setup.

The experimental setup is shown in Figure 4. The sensor was fabricated directly on the FC connector surface of one arm of a 2 × 2 3 dB coupler. The idle arm was tightly tied to suppress reflection. On the other side of the coupler, one arm was connected to a C+L band ASE light source and the other arm was connected to the optical spectrum analyzer (OSA, resolution 0.01 nm). The sensor head was placed into a test chamber (SETH-A-040U) with a RH resolution of 1% and temperature resolution of 0.1 °C. During the experiment, the temperature was tuned from 15 °C to 40 °C with a step of 5 °C. At each temperature, the RH was tuned from 20% to 90% with a step of 10% and stabilized for half an hour before the record of the spectrum. The temperature range is mainly restricted by the characteristics of the UV glue. We found in this range the sensor has good repeatability.

### 3.1. Humidity Response of the Sensor

**Figure 5:**
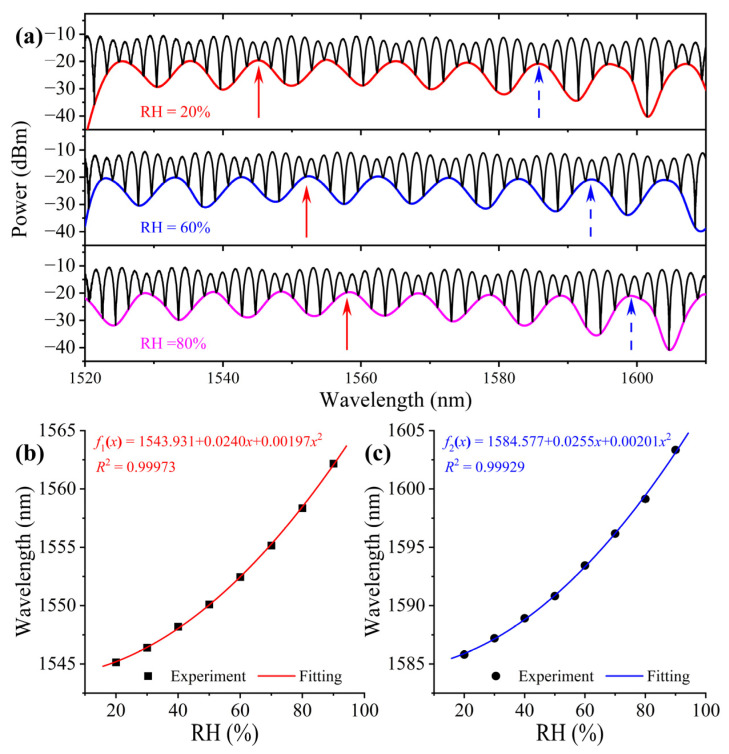
(**a**) Reflection spectra at different RH and the two peaks at the initial position of 1545.1 nm and 1585.8 nm at 30 °C pointed by red and blue arrows. (**b**,**c**) wavelength as function of RH of the two peaks.

As an example, Figure 5a shows spectra at RH values of 20%, 60%, and 80%, respectively, under the temperature of 30 °C. In the spectral range, there are plenty of peaks and dips on the envelope of the spectrum. Theoretically each of them can be tracked to demodulate the measurand. To investigate their difference, the peaks at 1545.1 nm and 1585.8 nm under the initial RH were examined. The wavelength of these peaks as a function of RH are plotted in Figure 5b,c, respectively. Firstly, we can see that the character wavelengths shift to the longer wavelength with the increase in RH. Secondly, the λ-RH relationship is nonlinear, which can be fitted very well with a quadratic polynomial. Thirdly, by taking the first-order derivative of the fitting equations shown in the figure, we can conclude: the sensitivity at a longer wavelength is higher than that at a shorter wavelength; and the RH sensitivity is linearly increasing with the RH. These features are common to other interferometric fiber sensors [24,25]. Thus, choosing a character wavelength at a longer wavelength is helpful to increase the sensitivity of the sensor. The highest RH sensitivities in the experimental range are 378.6 pm/%RH and 387.3 pm/%RH for the two peaks in the RH range.

### 3.2. Temperature Response of the Sensor

**Figure 6:**
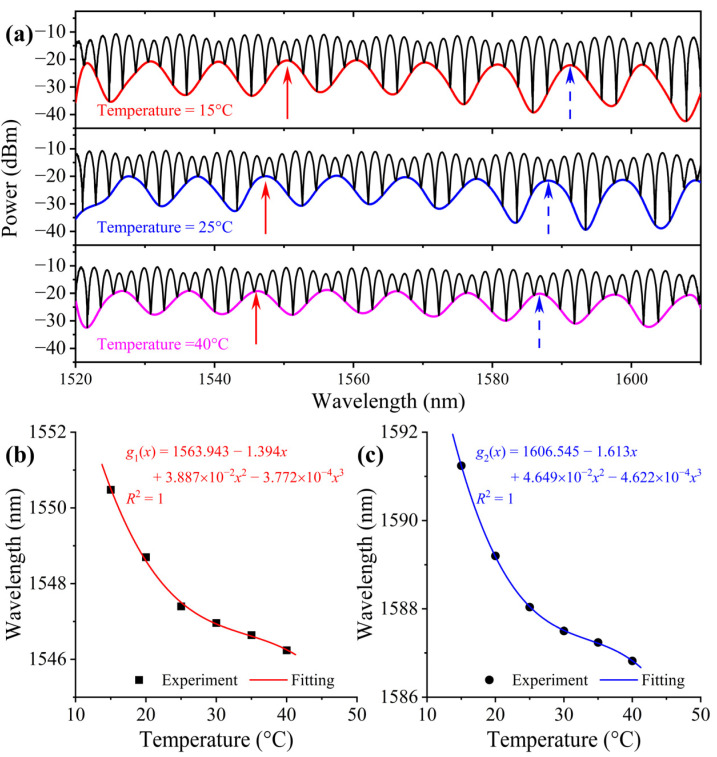
(**a**) Reflection spectra at different temperature and the two peaks at the initial position of 1550.5 nm and 1591.2 nm at 20%RH pointed by red and blue arrows. (**b**,**c**) wavelength as function of temperature of the two peaks.

**Figure 7:**
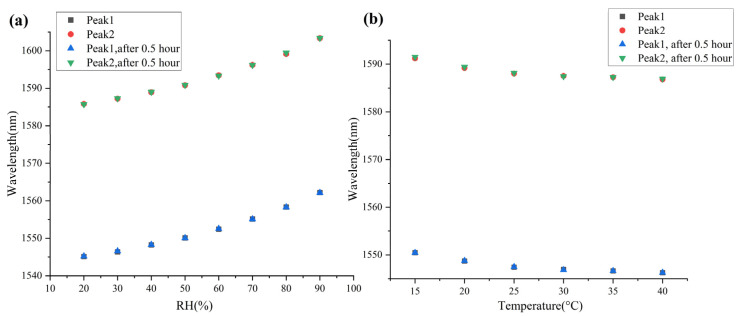
(**a**,**b**) Repeatability test of the sensor in this paper.

Figure 6a shows three typical spectra at 15 °C, 25 °C, and 40 °C, respectively, when the RH is fixed at 20%. From Figure 7 We can see that, contrary to the RH response of the sensor, a blue shift occurs when the temperature increases, that is the feature peaks shifts to the shorter wavelength end of the spectrum. Figure 6b,c are the λ-*T* relationship obtained by monitoring the two peaks, as indicated in Figure 7a. Obviously, the λ-*T* relationship is nonlinear. The temperature sensitivity of the longer wavelength peak is also higher than that of the shorter wavelength one. The curves can be perfectly fitted with a cubic polynomial. We believe the temperature response, as well as the RH response discussed previously, is closely related to the property of the UV glues selected to make the film. Thus, sensors with specific materials need to be calibrated specifically, but the working principle should be in common as long as the same design is used. The sensitivity of each peak at a given temperature can be calculated by taking the derivative of the corresponding fitting polynomial equation. Based on the experimental data shown in Figure 6b,c, the highest temperature sensitivities in the experimental range are −482.5 pm/°C and −533.0 pm/°C for the two peaks in the temperature range.

The comparison of the sensor in this paper with earlier reported sensors is listed in Table 1. It can be seen that the sensitivities of the sensor in this paper are higher than those of earlier reported sensors proposed for RH and temperature simultaneous measurement. Meanwhile, the RH measurement range of our sensor is much larger. In addition, the procedure of fabrication of our sensor is simple compared with other sensors, since it avoids splicing, chemical etching or other complicated processing procedure. These significant advantages make this sensor have a wide application potential compared to other sensors with the similar function. In addition, a repeatability test of the sensor is shown in Figure 7. Figure 7a,b show the results of re-measuring two peaks after 0.5 h in RH and temperature measurement, respectively. According to the comparison, the wavelengths of the two peaks are very stable. It can be considered that the small errors are caused by errors of measuring equipment.

**Figure 8:**
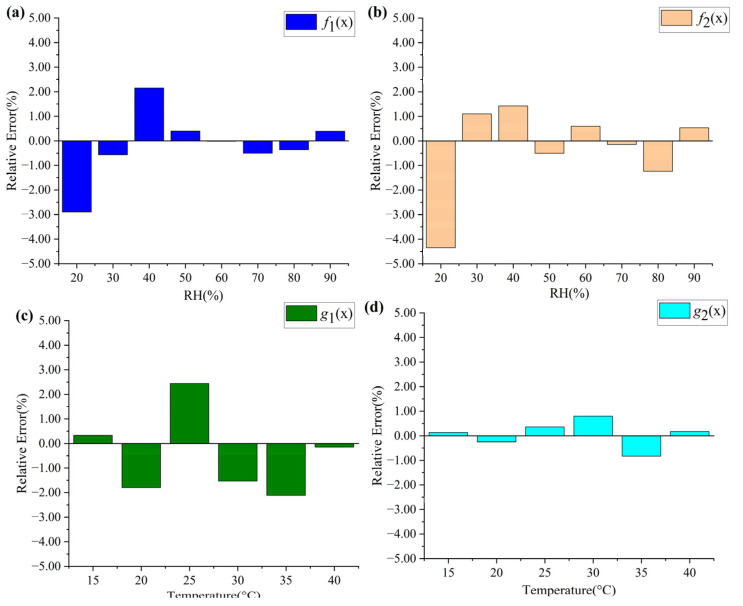
(**a**–**d**) Relative errors caused by fitting functions.

We also analyzed the relative errors caused by fitting. According to the fitting functions of RH and temperature, the RH and temperature were demodulated and compared with the experimental data, and the results are shown in Figure 8. It can be seen that the use of polynomial fitting leads to a relative error in the calculated results. Large relative errors generally occur when the measurand is small and the calculation results are within acceptable limits.

### 3.3. Simultaneous Measurement Method

Because both the humidity and temperature response of the sensor are nonlinear, as reveled by the above experimental results, it is required that two feature wavelengths be characterized in the calibration of the sensor, in order to demodulate the two measurands simultaneously. After calibration, fitting functions, as shown in Figure 6 and Figure 7, can be obtained. Then, the wavelength shifts of the two peaks under the combined influence of temperature and humidity can be expressed as:

(14) $$\begin{array}{l} \Delta \lambda_{1}=\left[f_{1}(20\%+\Delta \mathrm{RH})-f_{1}(20\%)\right]+\left[g_{1}(30{}^{\circ}C+\Delta T)-g_{1}(30{}^{\circ}C)\right],\\ \Delta \lambda_{2}=\left[f_{2}(20\%+\Delta \mathrm{RH})-f_{2}(20\%)\right]+\left[g_{2}(30{}^{\circ}C+\Delta T)-g_{2}(30{}^{\circ}C)\right], \end{array}$$

where 20% and 30 °C are the RH and temperature at which the sensor was calibrated. This is a set of two equations in two unknowns. With the measured $\Delta \lambda_{1}$ and $\Delta \lambda_{2}$, temperature and RH can be obtained by solving Equation (14). 

## 4. Conclusions

In conclusion, a fiber sensor for the simultaneous measurement of RH and temperature has been proposed and experimentally investigated in this paper. The sensor was fabricated by simply curing two kinds of polymer films with different refractive indexes and thicknesses on the end face of the FC path cord. The inner layer has a lower refractive index of 1.373 and a larger thickness of 369.9 µm, whereas the outer layer has a higher refractive index of 1.440 and a smaller thickness of 72.6 µm. The Vernier effect caused by the inner cavity and the whole cavity was utilized to improve the sensitivity of the sensor. Simultaneous temperature and relative humidity were accomplished by calibrating two feature wavelengths on the envelope of the reflection spectrum and then solving a set of nonlinear equations. For the sensor fabricated in this paper, the highest RH and temperature sensitivity achieved in the experimental range are 387.3 pm/%RH and −533.0 pm/°C, respectively. The sensor has the merits of simple in structure, easy of fabrication, and high sensitivity. It can find applications in monitoring RH and temperature of special environment, such as that of manned crafts, chemical reactions, and explosive warehouse, etc.

## Figures and Tables

**Table 1:** Comparison with other similar schemes.

Reference	Configuration	RH Range	RH Sensitivity	Temperature Range	Temperature Sensitivity
[15]	Two C-shaped FPIs	20%RH to 45%RH	−128 pm/%RH, 38 pm/%RH	15 °C to 45 °C	22 pm/°C, 722 pm/°C
[16]	coated POFBG	30%RH to 95%RH	11.8 pm/%RH, 13.7 pm/%RH	22 °C to 37 °C	−63.2 pm/°C, --
[17]	FPI+MZI	27.83%RH to 76.17%RH,	−132 pm/%RH, 72 pm/%RH	22.8 °C to 32.8 °C	172 pm/°C, 370 pm/°C
[18]	FPI+FBG	20%RH to 90%RH	22.07 pm/%RH, 0	15 °C to 65 °C	25.14 pm/°C, 9.98 pm/°C
This work	Two cascaded FPIs	20%RH to 90%RH	378.6 pm/%RH, 387.3 pm/%RH ^1^	15 °C to 40 °C	−482.5 pm/°C, 533.0 pm/°C ^2^

^1,2^ Highest sensitivities in the measurement range.

## Data Availability

Not applicable.
